# Awake Craniotomy for Eloquent Region Glioblastoma Classified by Tumor Location—A Retrospective and Prospective Cohort Study

**DOI:** 10.1002/cns.71126

**Published:** 2026-09-02

**Authors:** Yuzhe Li, Shimeng Weng, Jiahan Dong, Jiangwei Wang, Zhong Zhang, Xing Fan, Yinyan Wang, Wenbin Ma, Tao Jiang, Shengyu Fang

**Affiliations:** ^1^ Department of Neurosurgery The First Affiliated Hospital of Wenzhou Medical University Wenzhou China; ^2^ Department of Neurosurgery, Beijing Tiantan Hospital Capital Medical University Beijing China; ^3^ Beijing Neurosurgical Institute Capital Medical University Beijing China; ^4^ Chinese Academy of Medical Sciences and Peking Union Medical College Beijing China; ^5^ Research Unit of Accurate Diagnosis, Treatment, and Translational Medicine of Brain Tumors Chinese Academy of Medical Sciences Beijing China

**Keywords:** awake craniotomy, glioblastoma, prospective cohort, tumor location

## Abstract

**Introduction:**

The efficacy of awake craniotomy (AC) with intraoperative mapping for glioblastoma (GBM) in eloquent regions remains debated. This study aims to evaluate functional and survival outcomes of GBM patients undergoing AC stratified by tumor locations.

**Methods:**

A combined retrospective (2015–2023, *n* = 114: 43 AC vs. 71 standard craniotomy) and prospective cohort (2023–2025, *n* = 28: 13 AC vs. 15 standard craniotomy) of GBM patients with motor/language‐eloquent tumors was analyzed. Tumors were classified into motor subtypes (I: precentral gyrus; II: premotor/supplementary motor; III: internal capsule posterior limb; IV: other) and language subtypes (I: Broca's/precentral; II: postcentral/supramarginal gyrus; III: Wernicke's; IV: insular; V: other). Outcomes included extent of resection (EOR), postoperative motor/language recovery, overall survival (OS), and progression‐free survival (PFS).

**Results:**

The retrospective cohort demonstrated that AC has advantages in functional preservation across various motor/language subtypes. However, AC was associated with significantly deteriorated survival outcomes specifically in precentral gyrus GBMs. A prospective cohort study, enrolling only precentral gyrus GBMs for validation, yielded results consistent with the retrospective findings: worsened OS and PFS (OS: HR = 3.223, *p* = 0.0450; PFS: HR = 2.374, *p* = 0.0476); reduced EOR (AC: 74.3% ± 5.3%; standard craniotomy: 86.9% ± 12.3%, *p* = 0.0470); and better motor recovery.

**Conclusions:**

Functional preservation and survival outcomes of AC in GBM exhibited subtype‐specific correlations with tumor locations. AC with intraoperative mapping effectively preserves neurological function in GBM patients. However, for tumors involving the precentral gyrus, the AC approach carries greater risks than benefits and should be considered with caution.

**Trial Registration:**

Strategic Intervention on Preserving Motor Function During Awake Craniotomy: NCT05143788. Strategic Intervention on Preserving Language Function During Awake Craniotomy: NCT05143775

## Graphical abstract

Awake craniotomy preserves neurological function in eloquent glioblastomas, but for precentral gyrus tumors, it may reduce resection extent and impair survival, highlighting a location‐dependent risk–benefit balance.

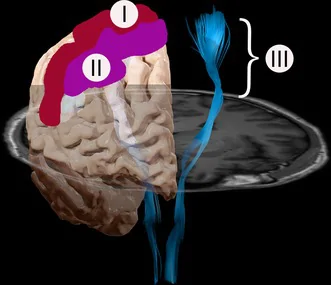

**Abbreviations**
AC: Awake Craniotomy
DTI: Diffusion Tensor Imaging
EOR: Extent of Resection
FMA: Fugl‐Meyer Assessment
GBM: Glioblastoma
GTR: Gross Total Resection
HR: Hazard Ratio
KPS: Karnofsky Performance Status
LGG: Low‐Grade Glioma
MRI: Magnetic Resonance Imaging
OS: Overall Survival
PFS: Progression‐Free Survival
pSLF/AF: posterior Superior Longitudinal Fasciculus/Arcuate Fasciculus
SLF/AF: Superior Longitudinal Fasciculus/Arcuate Fasciculus
SMA: Supplementary Motor Area

## 1 Introduction

Glioblastoma (GBM) is the most common primary malignant brain tumor [1]. In the period between 2007 and 2011, the incidence of glioblastoma was 1–4/100,000/year in China and 4.64/100,000/year in England [2, 3]. Maximal safe debulking has been advocated as the primary treatment for GBM [4].

Surgical guidelines of GBM prioritize maximal safe debulking correlated with the best overall prognosis [5]. Evidence indicates at least 70%–80% resection of the radiologically enhancing component benefits the survival of GBM [6, 7].

Although gross total resection (GTR) is associated with superior survival [8, 9], the infiltrative growth patterns of GBM cause challenges to GTR of GBM in eloquent regions. In these cases GTR could lead to extra risks of post‐operative neurological deficit, such as paralysis and aphasia. Intraoperative cortical and subcortical mapping techniques during Awake craniotomy (AC) have emerged for optimizing the resection extent of glioma in eloquent regions [10]. Current UK National Institute for Health and Care Excellence guidance protocols advise preoperative counseling regarding AC for eligible patients. Meta‐analysis studies about AC for glioma proved the neurophysiological mapping during AC significantly outperformed standard craniotomy in glioma, highlighting their capacity of optimizing the resection extent of glioma through real‐time functional feedback [11, 12]. In general, these studies did not distinguish between GBM and low‐grade glioma (LGG), but in clinical practice, there are significant differences in the treatment plans and surgical strategies between GBM and LGG; such heterogeneity obscures accurate assessment of AC's specific therapeutic value in GBM populations. GBM is characterized by poor prognosis and a high recurrence rate [13]. The balance between functional protection and achieving GTR in GBM patients requires more detailed criteria.

Currently, no classification protocol exists to assist neurosurgeons in risk stratification of AC for GBM. A preoperative classification system for assessing the feasibility and necessity of AC for GBM remains to be established. A meta‐analysis of AC observed that a supratentorial GBM cohort reached an acceptable rate of GTR, with reduced persistent neurological deficits [14], however, these studies did not stratify GBM subtypes or investigate more specific, location‐based surgical strategies.

Our previous research has developed an eloquent region glioma classification system based on tumor location that was useful for predicting postoperative motor and language functional prognosis in patients who underwent AC [15, 16]. We hypothesized that this glioma classification system could also assist neurosurgeons in the risk stratification of AC for GBM.

## 2 Method

### 2.1 Patients

The single‐centered retrospective study data was collected from 114 patients (43 underwent AC; 71 underwent standard craniotomy) with GBM involving motor‐related or language‐related eloquent regions, who underwent AC from 2015 to 2023 at the Glioma Treatment Center at an academic medical center in China. The inclusion criteria were as follows: (1) tumor located in motor‐related or language‐related eloquent areas or subcortical structures; (2) no uncontrolled systemic diseases, consciousness disorders, psychiatric disorders or other contraindications for awakening anesthesia; (3) age ≥ 18 years; (4) no history of neurosurgical surgery or radiotherapy; (5) without preoperative paralysis or aphasia; (6) preoperative KPS score was equal or greater than 90. Ethical approval for this retrospective study was received from the Local Institutional Review Board of the Hospital. It should be noted that the sample size in some subgroups was limited, which may affect statistical power.

The single‐centered prospective cohort study enrolled 28 GBM patients (13 underwent AC; 15 underwent standard craniotomy) from January 2023 to January 2025 at the Glioma Treatment Center at an academic medical center in China. The flow of participant enrollment is depicted in Figure S1. This cohort was designed as a targeted validation study to verify the specific findings from the retrospective analysis. Although the sample size is modest and precludes definitive conclusions, it serves to assess the consistency and reproducibility of our initial observations. The sample size was estimated based on our previous studies [16]. The inclusion criteria were as follows: (1) tumor involving the precentral gyrus; (2) no uncontrolled systemic diseases, consciousness disorders, psychiatric disorders or other contraindications for awakening anesthesia; (3) age ≥ 18 years; (4) no history of neurosurgical surgery or radiotherapy; (5) without preoperative paralysis; (6) preoperative KPS score was equal to or greater than 90. The surgical method is first determined based on the patient's will, then a senior neurosurgeon (one of the authors) determined the appropriate surgical method. Written consent was acquired from all patients, and clinical data were systematically evaluated. This study has been reported in line with the STROCSS criteria [17].

### 2.2 Tumor Classification: Motor‐Related Tumor Subtyping

Based on radiological observations and clinical experience, four distinct motor‐related tumor subtypes were categorized according to tumor location in our previous study [16] (Figure S2A–C): (I) Tumors involving the precentral gyrus; (II) Tumors involving premotor or supplementary motor area (SMA) without precentral gyrus infiltration; (III) Tumors adjacent to or invading the posterior limb of the internal capsule, defined as ≤ 5 mm proximity to the corticospinal tract at this level on diffusion tensor imaging (DTI). Distance quantification utilized coordinate‐based measurements; (IV) Supratentorial tumors not involving the above anatomical structures. Hierarchical classification prioritized Subtypes I–III when multiple motor‐related areas were involved. Tumor localization was assessed using T2‐FLAIR and contrast‐enhanced T1‐weighted MRI sequences.

### 2.3 Tumor Classification: Language‐Related Tumor Subtyping

Five additional subtypes were defined for language‐eloquent regions according to tumor location in our previous study [15](Figure S3A–C): (I) Tumors involving inferior frontal lobe (classical Broca's area) or precentral gyrus; (II) Tumors involving posterior central gyrus or supramarginal gyrus lesions superior to the lateral fissure; (III) Tumors involving the posterior region of the superior or middle temporal gyri (classical Wernicke's area) or the supramarginal gyrus (below the lateral fissure level); (IV) predominantly insular lobe infiltration; (V) Supratentorial tumors not involving the above anatomical structures. For multifocal language‐area tumors, concurrent insular involvement classified lesions as Type IV. Otherwise, classification followed the maximally invaded region. Type I, II, and III tumors were involved in the anterior segments, posterior portions, and inferior divisions of the superior longitudinal fasciculus/arcuate fasciculus (SLF/AF), respectively. Tumor localization was assessed using T2‐FLAIR and contrast‐enhanced T1‐weighted MRI sequences.

### 2.4 Awake Craniotomy Protocol

AC procedures were conducted by a senior neurosurgeon (one of the authors) with over 15 years of experience in AC. Cortical and subcortical mapping was conducted by Ojemann cortical stimulators (Radionics, USA) with standardized parameters (1–4 mA intensity, 60 Hz frequency, 1‐s square‐wave pulses). Positive motor responses were identified as involuntary hand/finger movements during stimulation of motor‐related structures (primary motor cortex and subcortical structures) or movement cessation during stimulation. For language monitoring, we set the stimulation intensity according to the threshold established for the M1 (primary motor cortex) area and then applied continuous stimulation to the language regions. Testing was performed using a picture‐naming task. The patient was asked to say, “This picture is…” (in Chinese, since all patients were Chinese). Each stimulation site was stimulated for 4 s. Positive language responses were identified as errors, pauses, and dysarthria during a continuous naming task upon stimulation of language‐related structures (Broca's area, Wernicke's area, and their subcortical structures). Each site underwent three stimulations; consistent positive reactions (≥ 2/3 trials) were marked with 5‐mm circular markers. Tumor resection adhered to navigational guidance (BrainLab neuronavigation) while the tumor resection was under the guidance of circular markers, instant function monitoring, and neuro‐navigation system (BrainLab, Munich, Germany). Critical vasculatures were preserved by using engraving resection. If uncontrollable seizures occur during intraoperative mapping, or if seizures recur for a second time, or if the patient requests conversion to general anesthesia due to discomfort, the procedure is immediately converted to general anesthesia, and the patient is excluded from the AC cohort.

Additionally, in motor‐related cases, intraoperative monitoring of motor evoked potentials was adopted to delineate the central sulcus and motor cortex areas in both AC procedures and non‐AC procedures.

### 2.5 Data Acquisition

For retrospective study, demographic, clinical, and pathological characteristics were collected from inpatient records. For cohort study, demographic and clinical characteristics were collected at the time of enrollment. Motor and language function was assessed preoperatively and at 3‐day, 14‐day, and 3‐month after AC. Motor function was assessed using the muscle strength test of the UK Medical Research Council, and language function was evaluated using the Western Aphasia Battery (WAB). Telephone follow‐ups records supplemented the recovery and prognosis data. A small number of samples overlapped with those used in our center's previously published studies [15], 13 GBM cases were reported before, accounting for 11.4% of all GBM cases. Patients were stratified into recovery groups based on whether the postoperative function returning to preoperative status. Preoperative magnetic resonance imaging (MRI) T2‐FLAIR images were used to calculate tumor volume. The tumor volumes were calculated in BrainLab neuro‐navigation system. EOR was calculated using preoperative and postoperative MRI scans with the following formula:

$$\mathrm{EOR}=\frac{\text{Preoperative volume}-\text{Postoperative volume}}{\text{Peroperative volume}.}$$

### 2.6 Statistical Analysis

Statistical analysis utilized GraphPad Prism 7 and SPSS Statistics 26 software. Parametric (two‐tailed Student's *t*‐test) and nonparametric (Mann–Whitney U) tests compared continuous variables. Categorical analyses employed chi‐square test or Fisher's exact test. Statistical significance was defined as **p < 0.05.

## 3 Results

Between 2015 and 2023, a total of 43 patients with GBM (25 male; 18 female) underwent AC with an average age of 43.6 years (range: 19–66), and 71 patients with GBM (42 male; 29 female) met the criterion of AC but underwent standard craniotomy with an average age of 48.1 years (range: 19–72) were included in the retrospective study. Demographic, clinical, and pathological characteristics of the AC group and standard craniotomy group are present in Table S1, and no significant difference was found in age, tumor volume, EOR, gender, MGMT promoter methylation, IDH status, 1p/19q status, and adjuvant therapy condition between the AC group and standard craniotomy group. Subtyping based on tumor location is shown in Table 1. We also performed baseline comparisons across subgroups, which similarly revealed no significant differences in these characteristics. We also performed subgroup analyses, which similarly revealed no significant differences in Demographic, clinical, and pathological characteristics Demographic, clinical, and pathological characteristics. We also performed subgroup analyses, which similarly revealed no significant differences in these characteristics (Table S2–S10). Language Type III and language Type IV cases didn't proceed with further analysis because their AC groups had less than 5 cases.

**TABLE 1:** Tumor classification: Motor/language‐related tumor subtyping.

Tumor classification	Awake craniotomy group	Standard craniotomy group
Motor classification		
I	15	14
II	9	13
III	7	12
IV	12	32
Language classification		
I	6	14
II	15	17
III	3	15
IV	4	12
V	15	13
Total count	43	71

*Note:* Motor‐related Tumor Subtyping: (I) precentral gyrus; (II) premotor or supplementary motor regions without precentral gyrus infiltration; (III) posterior limb of the internal capsule (IV) other. Language‐related Tumor Subtyping: (I) inferior frontal lobe/precentral gyrus; (II) posterior central gyrus/supramarginal gyrus lesions superior to the lateral fissure; (III) posterior region of the superior or middle temporal gyri or the supramarginal gyrus (below the lateral fissure level); (IV) insular lobe; (V) other. The shade is merely used to visually separate the two classification methods from each other.

After the review of the retrospective study outcomes, we managed to enroll a prospective cohort of Motor Type I cases to verify significant results in retrospective study. Between 2023 and 2025, a total of 13 patients with GBM underwent AC and 15 patients with GBM meeting the criterion of AC but undergoing standard craniotomy were enrolled in the prospective cohort. Demographic, clinical, and pathological characteristics had no significant difference between the AC group and the standard craniotomy group (Table S11).

### 3.1 Motor‐Related Tumor Subtyping Retrospective Outcomes

**FIGURE 1:**
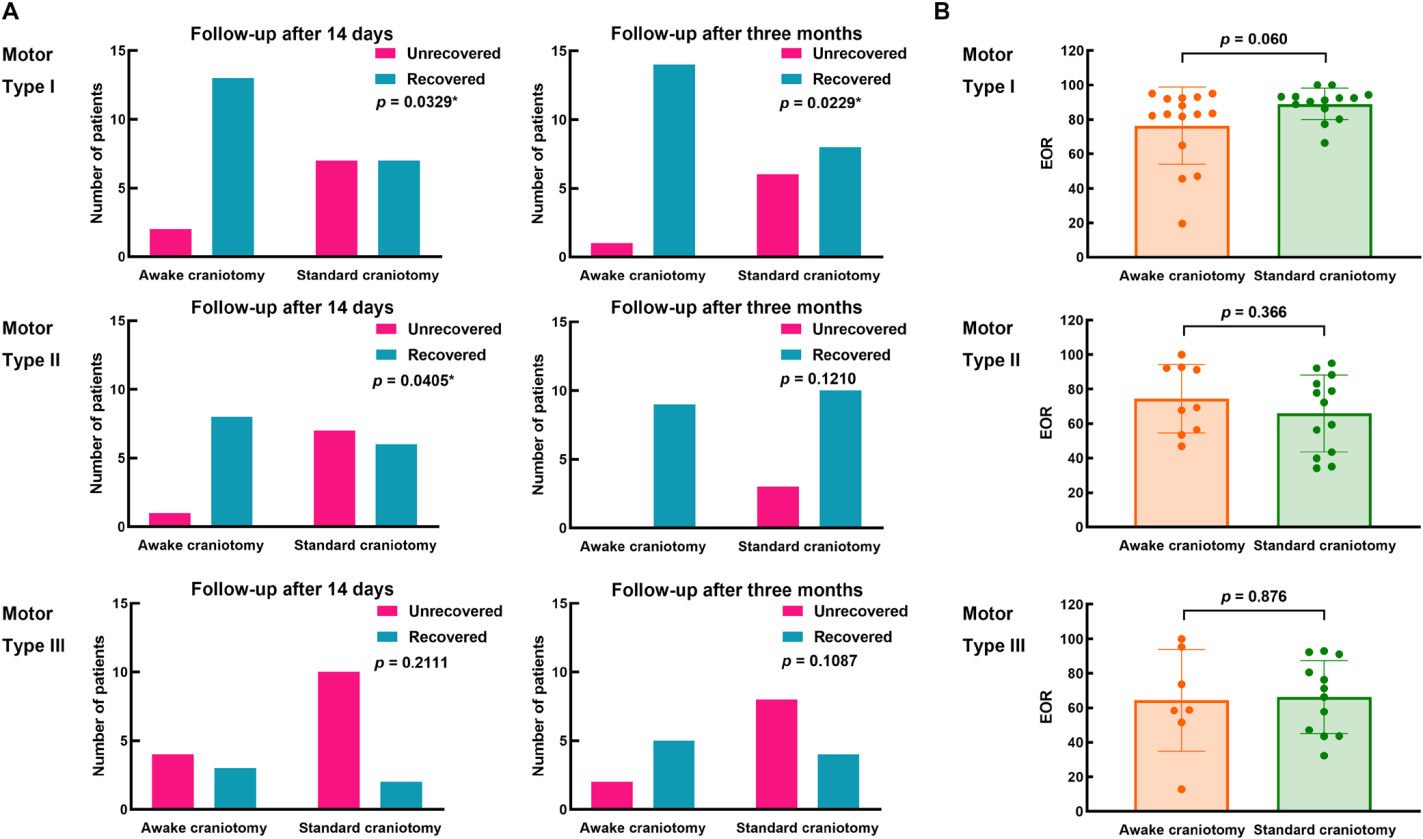
Functional recovery and extent of resection (EOR) of Motor‐related tumor subtypes. (A) Functional recovery at 14 days and 3 months after surgery of three subtypes; (B) EOR of three subtypes.

More than half of these patients experienced motor impairments or motor dysfunction within 1 week after operation in both AC group and standard craniotomy group. Since functional impairments in the acute postoperative period may be caused by edema of the surgical area, and this type of motor dysfunction can recover relatively quickly after the edema subsides, the motor function of 2 weeks and 3 months after the operation were chosen as the motor functional prognostic indicators (Figure 1A). In motor Type I cases, the AC group showed significant preferable motor function recovery rate at 2 weeks (AC:86.7%; standard craniotomy:50%, *p* = 0.0329) and 3 months (AC: 93.3%; standard craniotomy: 57.1%, *p* = 0.0229) after the operation. In motor Type II cases, the AC group showed significant preferable motor function recovery rate at 2 weeks after the operation (AC:88.9%; standard craniotomy:46.2%, *p* = 0.0405), and the result at 3 months after the operation were preferable but not significant (AC: 100%; standard craniotomy: 77.0%, *p* = 0.1210) after the operation. In motor Type III cases, the AC group showed preferable but not significant motor function recovery rate at 2 weeks (AC:42.9%; standard craniotomy:16.7%, *p* = 0.2111) and 3 months (AC: 71.4%; standard craniotomy: 33.3%, *p* = 0.1087). Among all motor‐related subtypes, there was no significant difference in the comparison of EOR between AC group and standard craniotomy group (Figure 1B), but no significant EOR was observed in the AC group among Type I motor cases (AC: 76.4% ± 5.8%; standard craniotomy: 89.0% ± 9.1%, *p* = 0.0596).

**FIGURE 2:**
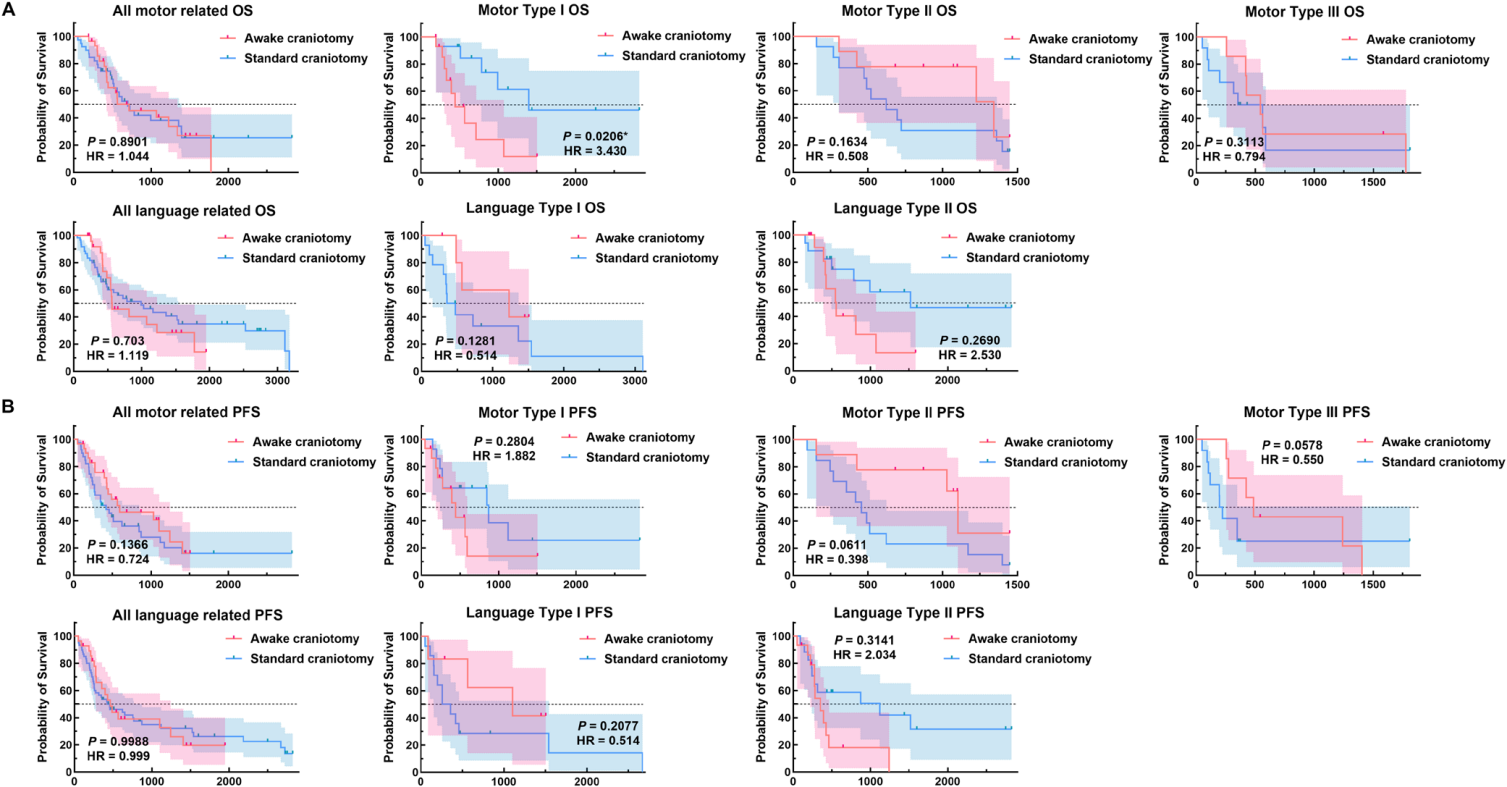
Kaplan–Meier Survival curve of all subtypes. (A) Overall survival (OS); (B) Progression‐free survival (PFS).

For all motor cases (Figure 2A,B), no significant difference of OS or PFS was found between AC group and standard craniotomy group (OS: HR = 1.044, *p* = 0.8901; PFS: HR = 0.724, *p* = 0.1366). For motor Type I tumors, AC is associated with significantly worse OS (HR = 3.430, *p* = 0.0206) and statistically insignificant worse PFS (HR = 1.882, *p* = 0.2804). For Motor Types II tumors and Motor Types III tumors, preferable but not significant PFS with AC was observed (Types II: HR = 0.398, *p* = 0.0611; Types III: HR = 0.350, *p* = 0.0578). Overall survival did not differ significantly between the two groups in Motor Types II and Motor Types III tumors.

### 3.2 Motor‐Related Tumor Subtyping Retrospective Outcomes

**FIGURE 3:**
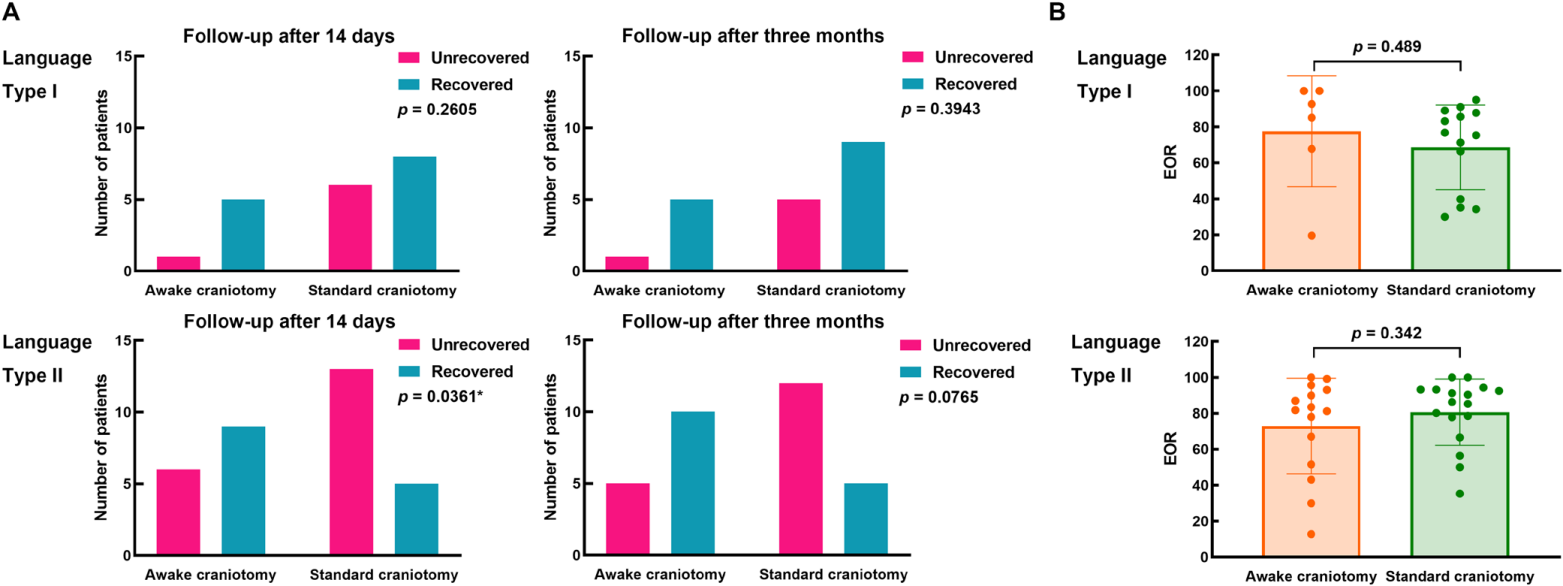
Functional recovery rate and extent of resection (EOR) of Language‐related tumor subtypes. (A) Functional recovery rate at 14 days and 3 months after surgery of Types I and II; (B) EOR of Types I and II.

In language Type I cases, the AC group showed a preferable but not significant language function recovery rate at 2 weeks (Figure 3A) (AC:83.3%; standard craniotomy:57.1%, *p* = 0.2605) and 3 months (AC: 83.3%; standard craniotomy: 64.3%, *p* = 0.3943) after the operation. In language Type II cases, the AC group showed a significant preferable language function recovery rate at 2 weeks after the operation (AC:60.0%; standard craniotomy:23.5%, *p* = 0.0361), and the result at 3 months after the operation was preferable but not significant (AC: 66.7%; standard craniotomy: 29.4%, *p* = 0.0765) after the operation. Among all language‐related subtypes, there was no significant difference in the comparison of EOR between the AC group and the standard craniotomy group (Figure 3B).

For all language cases (Figure 2A,B), no significant difference of OS or PFS was found between AC group and standard craniotomy group (OS: HR = 1.119, *p* = 0.703; PFS: HR = 0.999, *p* = 0.9988). For Language Type I tumors, no significant difference in OS and PFS was found between the two groups (OS: HR = 0.514, *p* = 0.1281; PFS: HR = 0.514, *p* = 0.2077). For Language Type II tumors, no significant difference in OS and PFS was found as well (OS: HR = 2.530, *p* = 0.2690; PFS: HR = 2.034, *p* = 0.3141).

### 3.3 Motor Type I Prospective Cohort Outcomes

**FIGURE 4:**
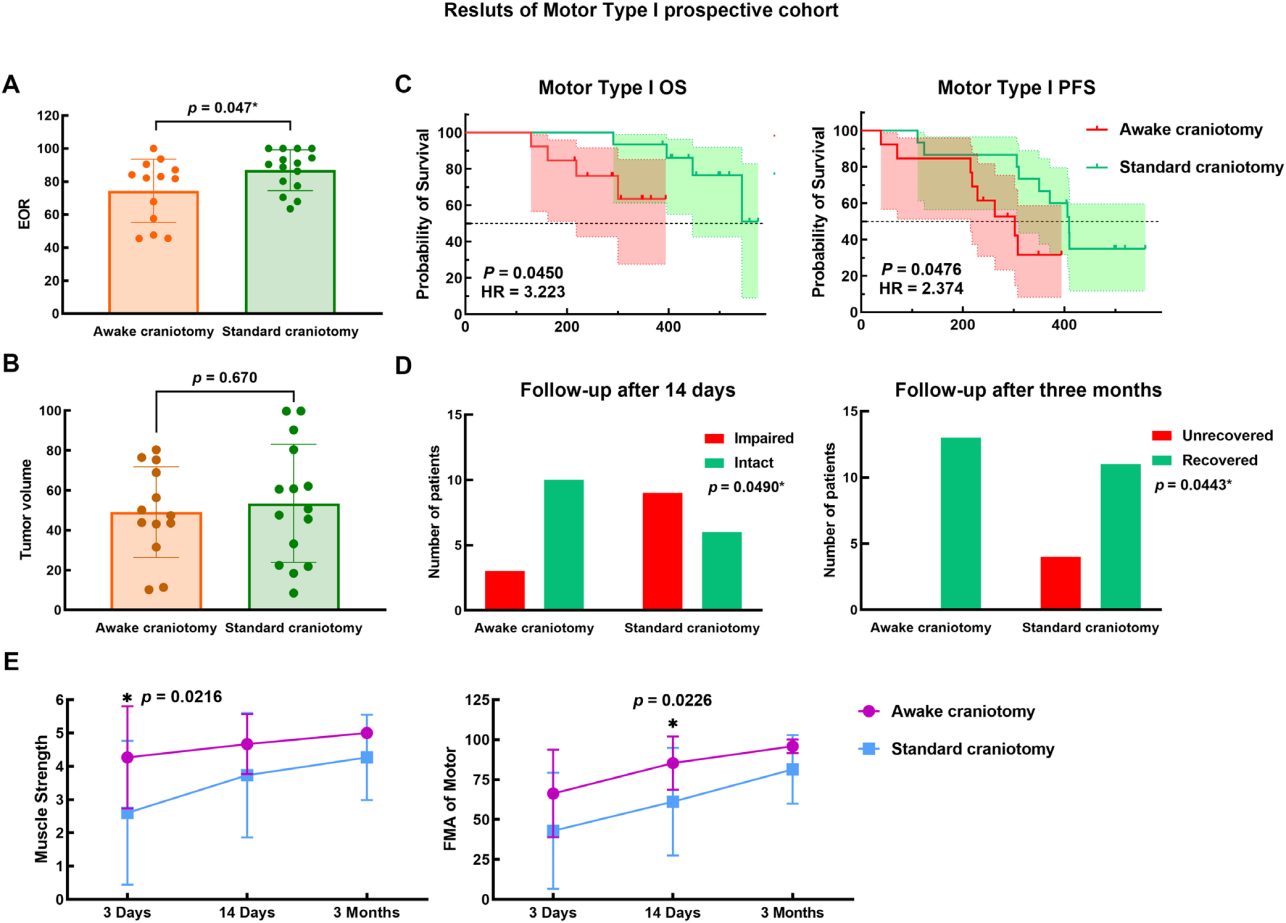
Motor Type I prospective cohort outcomes. (A) EOR difference between AC group and standard craniotomy group; (B) Tumor volume difference between AC group and standard craniotomy group; (C) Kaplan–Meier Survival curve of prospective cohort; (D) Functional recovery rate of prospective cohort; (E) Motor functional Assessment of prospective cohort (Left: Muscle strength level; Right: Fugl‐Meyer Assessment motor scores).

This cohort represents a validation of the Motor Type I analysis results, rather than a comprehensive validation of the tumor classification system. EOR exhibited a statistically significant reduction in the AC group (AC: 74.3% ± 5.3%; standard craniotomy: 86.9% ± 12.3%, *p* = 0.0470) with tumor volume having no significant difference (Figure 4A,B). Meanwhile, OS and PFS of AC cohort were significantly worse (Figure 4C) (OS: HR = 3.223, *p* = 0.0450; PFS: HR = 2.374, *p* = 0.0476).

For functional Outcomes (Figure 4D,E), AC cohort was associated with significantly better postoperative function recovery rate at both postoperative 2 weeks (*p* = 0.0490) and 3 months (*p* = 0.0443). Fugl‐Meyer Assessment (FMA) motor scores were significantly better at 14 days after surgery (AC: 85.4 ± 16.7; standard craniotomy: 61.2 ± 33.6, *p* = 0.0226, Table S12), and muscle strength was significantly better at 3 days after surgery (AC: 4.2 ± 1.6; standard craniotomy: 2.6 ± 2.2, *p* = 0.0437, Table S13).

**FIGURE 5:**
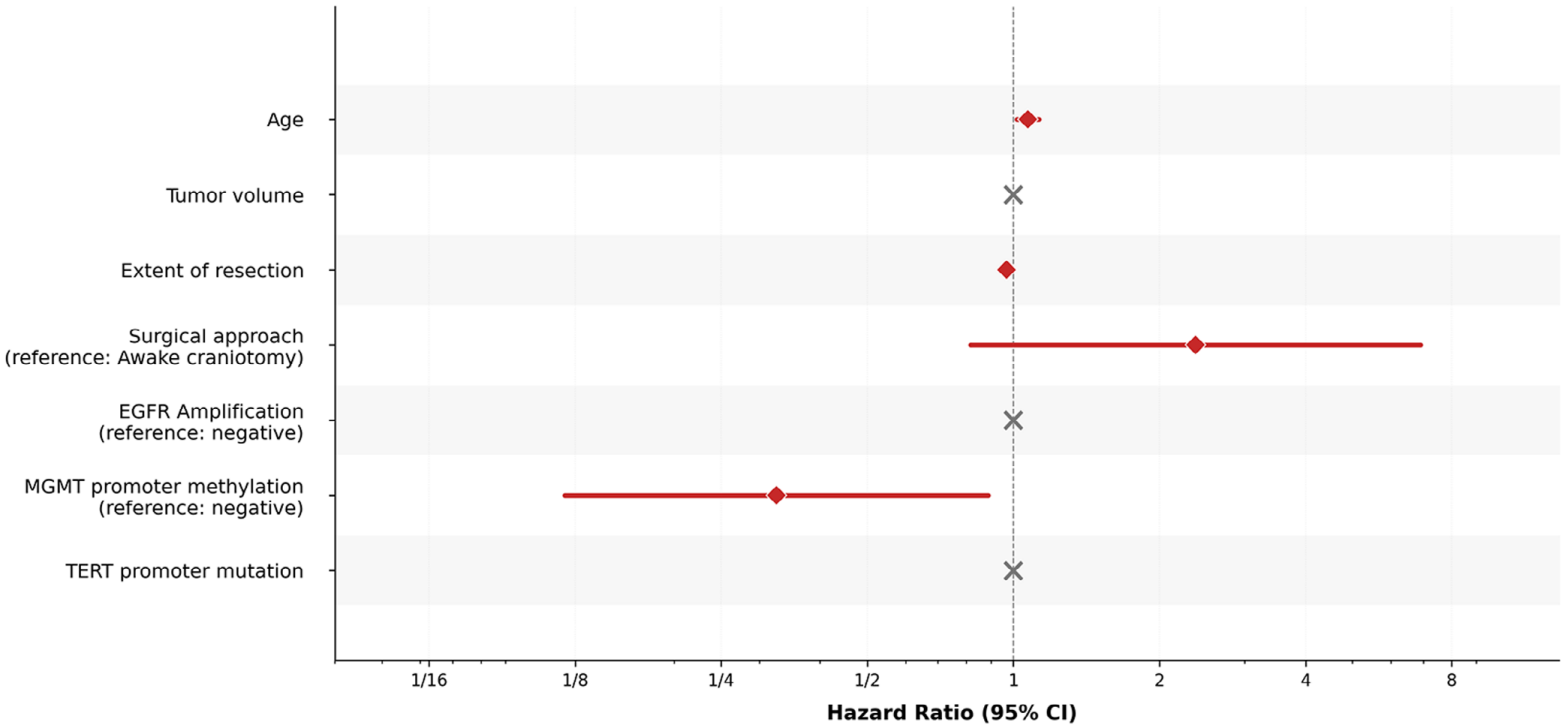
Univariate Cox regression analysis of progression‐free survival in prospective cohort.

Multivariable Cox regression (forward, LR) of motor Type I prospective cohort identified no independent predictors for overall survival (Table S14). In univariate regression analysis for progression‐free survival, age, extent of resection, surgical approach, and MGMT promoter methylation were significant and thus entered into multivariate regression analysis (Figure 5). The multivariate regression analysis revealed older age (HR = 1.135 per year, 95% CI: 1.043–1.236, *p* = 0.003) was an independent risk factor, while greater extent of resection (HR = 0.962 per percent, 95% CI: 0.925–1.001, *p* = 0.046) and MGMT promoter methylation (HR = 0.096, 95% CI: 0.019–0.474, *p* = 0.004) were independent protective factors. The surgical approach was not an independent predictor of OS (*p* = 0.327) or PFS (*p* = 0.261) (Table 2).

**TABLE 2:** Multivariable Cox regression analysis of progression‐free survival in prospective cohort.

Demographic and clinical characteristics	Univariate analysis			Multivariate analysis		
	*p*	Hazard ratio	95% CI	*p*	Hazard ratio	95% CI
Age	0.012*	1.071	1.015 to 1.130	0.001*	1.133	1.053 to 1.220
Tumor volume	0.943	—	—	—	—	—
Extent of resection	0.036*	0.969	0.942 to 0.998	0.036*	0.963	0.930 to 0.998
Surgical approach (reference: Awake craniotomy)	0.048*	2.374	0.816 to 6.904	0.806	—	—
EGFR amplification (reference: negative)	0.449	—	—	—	—	—
TERT promoter mutation	0.306					
MGMT promoter methylation (reference: negative)	0.028*	0.325	0.119 to 0.887	0.027*	0.227	0.061 to 0.845

*Note:* Multivariate analysis method: forward, LR.

* *p* < 0.05.

## 4 Discussion

AC with intraoperative mapping remains an established strategy for functional protection during tumor resection [18]. Unlike LGG, robust literature has validated maximal safe debulking as a cornerstone of high‐grade glioma management (including GBM), so balancing functional protection and achieving the GTR rate in GBM is crucial [6, 7, 19, 20]. A Japanese trial (30 AC; 30 standard craniotomy) identified preoperative KPS ≥ 90 as independent predictors of prognosis in GBM AC [21], so our study only included cases with KPS ≥ 90. This single‐center study delineates the experience of AC in GBM surgery, with tumor subtyping stratified according to the relationship between tumor localization and eloquent regions. The results based on the retrospective study (43 AC; 71 standard craniotomy) are consistent with previous studies at the unstratified level [12, 18, 22]. However, further conclusions were reached after subtyping according to tumor location.

### 4.1 Motor‐Related GBM Classification

Tumors involving M1 (Type I), SMA (Type II) and the posterior limb of the internal capsule (Type III) were defined as motor‐related GBM. Functional preservation and survival outcomes of AC in GBM exhibited subtype‐specific characteristics. Regarding motor dysfunction recovery, the results of the retrospective study demonstrate the protective effects of AC on neurological function in motor‐related GBMs, which is consistent with findings reported in previous literature [12, 18, 22]. Tumors involving SMA (Type II) predominantly manifested reversible deficits consistent with SMA syndrome; permanent impairment of muscle strength is infrequent [23]. This subtype demonstrated improvements in both survival outcomes and functional recovery following AC surgery. Tumors involving the posterior limb of the internal capsule (Type III) correlated with corticospinal tract ischemia or injury [24, 25]. This subtype demonstrated higher rates of permanent motor impairment compared to other anatomical subgroups; this result is consistent with our previous study regarding glioma [26]. Notably, tumors involving the precentral gyrus (Motor Type I) have significantly worse OS, a finding that has never been reported in previous literature. Given the inconsistency between the survival outcomes of tumors involving the precentral gyrus observed in our study and those reported in previous research, we further validated the findings by collecting a prospective cohort of GBM cases with precentral gyrus involvement. In the prospective cohort, the AC group demonstrated significant superiority in motor function preservation and worse OS/PFS. Multivariable analysis confirmed that EOR was an independent predictor of PFS. This suggests that for precentral gyrus GBMs, the survival disadvantage observed with AC is not direct, but mediated through a reduction in EOR due to functional preservation constraints. The observed worse survival in AC for motor Type I tumors may be mediated by reduced extent of resection, rather than by AC itself. M1 tumor (Type I) location determines the displacement of cortical hand representation, and resection of tumor in the precentral gyrus carries a risk of severe motor dysfunction [27, 28], the protective effect of AC on motor function was significant after surgery, while at the 3‐month postoperative follow‐up, there was no significant difference in FMA scores between the AC group and the group that underwent intraoperative monitoring of motor evoked potentials without AC. Therefore, for Motor Type I GBMs, AC should not be performed at the expense of the resection rate; otherwise, the decision to perform AC requires careful weighing of its functional benefits against the potential risk of compromising the resection rate.

### 4.2 Language‐Related GBM Classification

In language‐related cases, tumors involving Inferior frontal lobe (Broca's area) or precentral gyrus (Type I) tumors have preferable but not significant OS and PFS with AC, while the EOR of the AC group is relatively higher. Our previous research identified gliomas involving posterior central gyrus or supramarginal gyrus lesions superior to the lateral fissure and lesions adjacent to the posterior segment of SLF/AF (Type II) as having a high incidence of permanent postoperative language deficit [15], which is consistent with the results of this study. The mechanism underlying this phenomenon could be the significantly reduced functional reorganization capacity in the posterior segment of SLF/AF compared to its anterior and inferior parts [29]. SLF/AF fibers communicate between superior temporal, parietal, and inferior frontal regions [30, 31], so the protection of pSLF/AF fibers during surgery is of vital importance. The limited sample size for Types III and IV Language‐related GBMs in our study precluded a robust analysis. Therefore, further dedicated studies are required to investigate language‐related GBMs.

## 5 Limitations

First, this study is a single‐center design and non‐randomized surgical technique assignment may introduce selection bias, despite our efforts to match inclusion criteria. Although surgical approach selection was based on patient preference and surgeon judgment, which introduced selection bias in this study, we adopted this practice because it was in the patients' best interests. Second, the sample size, particularly within certain tumor subtypes, was relatively small. This limits the robustness of our subgroup analyses and increases the risk of Type II errors, highlighting the need for future multicenter collaborations to fully validate the proposed classification system. Also, the literature has reported evidence for hemispheric specialization and ipsilateral control in the motor control system [32]. Due to the limited sample size, the present study did not perform a subgroup analysis of left‐ versus right‐sided tumors to explore tumor laterality; further studies are warranted.

## 6 Conclusion

In conclusion, functional preservation and survival outcomes of AC in eloquent region GBM exhibited subtype‐specific correlations with tumor locations. AC improves both survival and functional outcomes in general. However, for tumors involving the precentral gyrus, the AC approach carries greater risks of survival, it should not be performed at the expense of the resection rate, otherwise the decision to perform AC requires careful weighing of its functional benefits against the potential risk of compromising the resection rate.

## Author Contributions

Conception and design: Yuzhe Li, Shengyu Fang. Acquisition and analysis of data: Shengyu Fang, Yuzhe Li, and Zhong Zhang. Writing, review, and/or revision of the manuscript: Shengyu Fang, Yuzhe Li, Shimeng Weng, Jiahan Dong, Jiangwei Wang, Yinyan Wang, Xing Fan, Wenbin Ma. Study supervision: Tao Jiang, Wenbin Ma. The authors read and approved the final manuscript.

## Funding

This study was supported by funds as follows: National Natural Science Foundation of China (No. 82203170); Beijing Natural Science Foundation (No. JQ23040); Brain Tumor Precision Diagnosis and Treatment and Translational Medicine Innovation Unit, Chinese Academy of Medical Sciences (2019‐I2M‐5‐021).

## Ethics Statement

This study was conducted in accordance with the Declaration of Helsinki and was approved by the Ethics Committee of Beijing Tiantan Hospital (Approval No.: KY‐2022‐206‐03; Date: 2022/12/28).

## Consent

Written informed consent was obtained from all participants. The recruitment and data collection period spanned from January 2023 to January 2025.

## Conflicts of Interest

The authors declare no conflicts of interest.

## Supporting information


**Table S1:** Demographic and Clinical Characteristics of the Retrospective Study Population.
**Table S2:** Demographic and Clinical Characteristics of Motor Type I (Retrospective Study).
**Table S3:** Demographic and Clinical Characteristics of Motor Type II (Retrospective Study).
**Table S4:** Demographic and Clinical Characteristics of Motor Type III (Retrospective Study).
**Table S5:** Demographic and Clinical Characteristics of Motor Type IV (Retrospective Study).
**Table S6:** Demographic and Clinical Characteristics of Language Type I (Retrospective Study).
**Table S7:** Demographic and Clinical Characteristics of Language Type II (Retrospective Study).
**Table S8:** Demographic and Clinical Characteristics of Language Type III (Retrospective Study).
**Table S9:** Demographic and Clinical Characteristics of Language Type IV (Retrospective Study).
**Table S10:** Demographic and Clinical Characteristics of Language Type V (Retrospective Study).
**Table S11:** Demographic and Clinical Characteristics of the Cohort Study Population.
**Table S12:** Change in the Fugl–Meyer Assessment score in a prospective cohort.
**Table S13:** Change in the muscle strength in a prospective cohort.
**Table S14:** Multivariable Cox regression analysis of overall survival in prospective cohort.
**Figure S1:** Flow diagram of the progress through the phases of a prospective cohort of GBM involving the precentral gyrus.
**Figure S2:** Motor‐related tumor subtypes diagram. A: Axial plane; B: Sagittal plane; C: Coronal plane.
**Figure S3:** Language‐related tumor subtypes diagram. A: Sagittal plane with brain mask; B: Sagittal plane showing insular lobe and Type IV; C: Sagittal plane showing Superior Longitudinal Fasciculus/Arcuate Fasciculus (SLF/AF) invasion in Types I, II, and III.

## Data Availability

The data that support the findings of this study are available from the corresponding author upon reasonable request.
