# Supplementary material for: Awake Craniotomy for Eloquent Region Glioblastoma Classified by Tumor Location—A Retrospective and Prospective Cohort Study
Source: CNS Neurosci Ther. 2026 Sep 2;32(9):e71126. doi: 10.1002/cns.71126 (PMC13538451; doi:10.1002/cns.71126)
Supplement: Supplementary file 1 [file CNS-32-e71126-s001.docx]

| **Supplementary Table 1. Demographic and Clinical Characteristics of the Retrospective Study Population** | | | | | |
| --- | --- | --- | --- | --- | --- |
| **Characteristics** | **Awake craniotomy group** | | | **Standard craniotomy group** | ***P* value** |
| **Gender** | |  |  | |  |
| Male | | 25 | 42 | | 0.915 |
| Female | | 18 | 29 | |  |
| **Age (years)** | | 43.6 ± 1.8 | 48.1 ± 1.6 | | 0.067 |
| **Tumor volume (ml)** | | 44.4 ± 4.6 | 53.3 ± 3.9 | | 0.133 |
| **Extent of resection (%)** | | 74.9 ± 2.4 | 77.6 ± 3.8 | | 0.527 |
| **MGMT promoter methylation** | |  |  | |  |
| Positive | | 17 | 31 | | 0.557 |
| Negative | | 19 | 27 | |  |
| **EGFR Amplification** | |  |  | |  |
| Positive | | 14 | 23 | | 0.782 |
| Negative | | 24 | 35 | |  |
| **TERT promoter mutation** | |  |  | |  |
| Positive | | 0 | 1 | | 0.884 |
| Negative | | 36 | 45 | |  |
| **Adjuvant therapy** | |  |  | |  |
| Yes | | 37 | 59 | | 0.676 |
| No | | 6 | 12 | |  |
| **Student's t-test**: Age, Tumor volume, Extent of resection. **chi-square/Fisher’s exact test:** Gender, MGMT promoter methylation, EGFR Amplification, TERT promoter mutation, Adjuvant therapy. The pathological results of some cases are missing. | | | | | |

| **Supplementary Table 2. Demographic and Clinical Characteristics of Motor type I (Retrospective Study)** | | | | | |
| --- | --- | --- | --- | --- | --- |
| **Characteristics** | **Awake craniotomy group** | | | **Standard craniotomy group** | ***P* value** |
| **Gender** | |  |  | |  |
| Male | | 11 | 8 | | 0.450 |
| Female | | 4 | 6 | |  |
| **Age (years)** | | 46.5 ± 2.9 | 50.0 ± 3.6 | | 0.462 |
| **Tumor volume (ml)** | | 44.7 ± 7.9 | 49.5 ± 8.6 | | 0.679 |
| **Extent of resection (%)** | | 83.4 ± 6.1 | 89.0 ± 9.0 | | 0.403 |
| **MGMT promoter methylation** | |  |  | |  |
| Positive | | 6 | 3 | | 0.400 |
| Negative | | 6 | 8 | |  |
| **EGFR Amplification** | |  |  | |  |
| Positive | | 3 | 2 | | 0.583 |
| Negative | | 10 | 3 | |  |
| **TERT promoter mutation** | |  |  | |  |
| Positive | | 6 | 6 | | ＞0.999 |
| Negative | | 6 | 5 | |  |
| **Adjuvant therapy** | |  |  | |  |
| Yes | | 14 | 14 | | ＞0.999 |
| No | | 1 | 0 | |  |
| **Student's t-test**: Age, Tumor volume, Extent of resection. **chi-square/Fisher’s exact test:** Gender, MGMT promoter methylation, EGFR Amplification, TERT promoter mutation, Adjuvant therapy. The pathological results of some cases are missing. | | | | | |

| **Supplementary Table 3. Demographic and Clinical Characteristics of Motor type II (Retrospective Study)** | | | | | |
| --- | --- | --- | --- | --- | --- |
| **Characteristics** | **Awake craniotomy group** | | | **Standard craniotomy group** | ***P* value** |
| **Gender** | |  |  | |  |
| Male | | 3 | 6 | | 0.674 |
| Female | | 6 | 7 | |  |
| **Age (years)** | | 39.1 ± 4.1 | 43.0 ± 4.4 | | 0.529 |
| **Tumor volume (ml)** | | 43.6 ± 13.1 | 59.1 ± 11.4 | | 0.381 |
| **Extent of resection (%)** | | 74.4 ± 6.6 | 65.8 ± 6.1 | | 0.357 |
| **MGMT promoter methylation** | |  |  | |  |
| Positive | | 4 | 5 | | ＞0.999 |
| Negative | | 4 | 5 | |  |
| **EGFR Amplification** | |  |  | |  |
| Positive | | 3 | 4 | | 0.619 |
| Negative | | 5 | 3 | |  |
| **TERT promoter mutation** | |  |  | |  |
| Positive | | 4 | 7 | | 0.630 |
| Negative | | 4 | 3 | |  |
| **Adjuvant therapy** | |  |  | |  |
| Yes | | 9 | 13 | | / |
| No | | 0 | 0 | |  |
| **Student's t-test**: Age, Tumor volume, Extent of resection. **chi-square/Fisher’s exact test:** Gender, MGMT promoter methylation, EGFR Amplification, TERT promoter mutation, Adjuvant therapy. The pathological results of some cases are missing. | | | | | |

| **Supplementary Table 4. Demographic and Clinical Characteristics of Motor type III (Retrospective Study)** | | | | | |
| --- | --- | --- | --- | --- | --- |
| **Characteristics** | **Awake craniotomy group** | | | **Standard craniotomy group** | ***P* value** |
| **Gender** | |  |  | |  |
| Male | | 4 | 8 | | ＞0.999 |
| Female | | 3 | 4 | |  |
| **Age (years)** | | 43.7 ± 3.5 | 48.5 ± 4.2 | | 0.390 |
| **Tumor volume (ml)** | | 54.6 ± 5.1 | 63.8 ± 8.9 | | 0.385 |
| **Extent of resection (%)** | | 64.4 ± 11.1 | 66.2 ± 6.1 | | 0.887 |
| **MGMT promoter methylation** | |  |  | |  |
| Positive | | 2 | 7 | | 0.329 |
| Negative | | 3 | 3 | |  |
| **EGFR Amplification** | |  |  | |  |
| Positive | | 3 | 1 | | ＞0.999 |
| Negative | | 2 | 0 | |  |
| **TERT promoter mutation** | |  |  | |  |
| Positive | | 3 | 7 | | ＞0.999 |
| Negative | | 2 | 3 | |  |
| **Adjuvant therapy** | |  |  | |  |
| Yes | | 7 | 10 | | 0.509 |
| No | | 0 | 2 | |  |
| **Student's t-test**: Age, Tumor volume, Extent of resection. **chi-square/Fisher’s exact test:** Gender, MGMT promoter methylation, EGFR Amplification, TERT promoter mutation, Adjuvant therapy. The pathological results of some cases are missing. | | | | | |

| **Supplementary Table 5. Demographic and Clinical Characteristics of Motor type IV (Retrospective Study)** | | | | | |
| --- | --- | --- | --- | --- | --- |
| **Characteristics** | **Awake craniotomy group** | | | **Standard craniotomy group** | ***P* value** |
| **Gender** | |  |  | |  |
| Male | | 7 | 20 | | ＞0.999 |
| Female | | 5 | 12 | |  |
| **Age (years)** | | 43.3 ± 3.7 | 49.2 ± 2.0 | | 0.172 |
| **Tumor volume (ml)** | | 37.8 ± 8.1 | 48.7 ± 5.2 | | 0.272 |
| **Extent of resection (%)** | | 80.5 ± 6.9 | 75.7 ± 3.5 | | 0.541 |
| **MGMT promoter methylation** | |  |  | |  |
| Positive | | 5 | 16 | | 0.491 |
| Negative | | 6 | 11 | |  |
| **EGFR Amplification** | |  |  | |  |
| Positive | | 3 | 3 | | ＞0.999 |
| Negative | | 7 | 9 | |  |
| **TERT promoter mutation** | |  |  | |  |
| Positive | | 6 | 3 | | 0.272 |
| Negative | | 5 | 9 | |  |
| **Adjuvant therapy** | |  |  | |  |
| Yes | | 10 | 29 | | 0.603 |
| No | | 2 | 3 | |  |
| **Student's t-test**: Age, Tumor volume, Extent of resection. **chi-square/Fisher’s exact test:** Gender, MGMT promoter methylation, EGFR Amplification, TERT promoter mutation, Adjuvant therapy. The pathological results of some cases are missing. | | | | | |

| **Supplementary Table 6. Demographic and Clinical Characteristics of** **Language type I (Retrospective Study)** | | | | | |
| --- | --- | --- | --- | --- | --- |
| **Characteristics** | **Awake craniotomy group** | | | **Standard craniotomy group** | ***P* value** |
| **Gender** | |  |  | |  |
| Male | | 1 | 8 | | 0.157 |
| Female | | 5 | 6 | |  |
| **Age (years)** | | 39.8 ± 6.4 | 40.4 ± 3.8 | | 0.938 |
| **Tumor volume (ml)** | | 41.0 ± 9.3 | 67.1 ± 9.6 | | 0.069 |
| **Extent of resection (%)** | | 77.5 ± 12.6 | 68.6 ± 6.3 | | 0.546 |
| **MGMT promoter methylation** | |  |  | |  |
| Positive | | 2 | 8 | | 0.299 |
| Negative | | 3 | 3 | |  |
| **EGFR Amplification** | |  |  | |  |
| Positive | | 0 | 0 | | / |
| Negative | | 5 | 3 | |  |
| **TERT promoter mutation** | |  |  | |  |
| Positive | | 4 | 9 | | ＞0.999 |
| Negative | | 1 | 2 | |  |
| **Adjuvant therapy** | |  |  | |  |
| Yes | | 5 | 12 | | ＞0.999 |
| No | | 1 | 2 | |  |
| **Student's t-test**: Age, Tumor volume, Extent of resection. **chi-square/Fisher’s exact test:** Gender, MGMT promoter methylation, EGFR Amplification, TERT promoter mutation, Adjuvant therapy. The pathological results of some cases are missing. | | | | | |

| **Supplementary Table 7. Demographic and Clinical Characteristics of Language type II (Retrospective Study)** | | | | | |
| --- | --- | --- | --- | --- | --- |
| **Characteristics** | **Awake craniotomy group** | | | **Standard craniotomy group** | ***P* value** |
| **Gender** | |  |  | |  |
| Male | | 9 | 7 | | 0.479 |
| Female | | 6 | 10 | |  |
| **Age (years)** | | 47.7 ± 2.0 | 53.6 ± 2.7 | | 0.090 |
| **Tumor volume (ml)** | | 42.9 ± 6.9 | 53.0 ± 9.9 | | 0.411 |
| **Extent of resection (%)** | | 78.9 ± 7.7 | 83.9 ± 4.6 | | 0.580 |
| **MGMT promoter methylation** | |  |  | |  |
| Positive | | 7 | 4 | | 0.345 |
| Negative | | 6 | 10 | |  |
| **EGFR Amplification** | |  |  | |  |
| Positive | | 5 | 3 | | ＞0.999 |
| Negative | | 9 | 6 | |  |
| **TERT promoter mutation** | |  |  | |  |
| Positive | | 5 | 9 | | 0.339 |
| Negative | | 8 | 5 | |  |
| **Adjuvant therapy** | |  |  | |  |
| Yes | | 15 | 17 | | / |
| No | | 0 | 0 | |  |
| **Student's t-test**: Age, Tumor volume, Extent of resection. **chi-square/Fisher’s exact test:** Gender, MGMT promoter methylation, EGFR Amplification, TERT promoter mutation, Adjuvant therapy. The pathological results of some cases are missing. | | | | | |

| **Supplementary Table 8. Demographic and Clinical Characteristics of** **Language type III (Retrospective Study)** | | | | | |
| --- | --- | --- | --- | --- | --- |
| **Characteristics** | **Awake craniotomy group** | | | **Standard craniotomy group** | ***P* value** |
| **Gender** | |  |  | |  |
| Male | | 3 | 13 | | ＞0.999 |
| Female | | 0 | 2 | |  |
| **Age (years)** | | 38.7 ± 9.3 | 50.7 ± 3.4 | | 0.324 |
| **Tumor volume (ml)** | | 42.5 ± 4.4 | 44.8 ± 6.7 | | 0.783 |
| **Extent of resection (%)** | | 65.7 ± 3.8 | 70.2 ± 4.9 | | 0.483 |
| **MGMT promoter methylation** | |  |  | |  |
| Positive | | 0 | 6 | | 0.462 |
| Negative | | 2 | 5 | |  |
| **EGFR Amplification** | |  |  | |  |
| Positive | | 1 | 1 | | ＞0.999 |
| Negative | | 1 | 2 | |  |
| **TERT promoter mutation** | |  |  | |  |
| Positive | | 2 | 9 | | ＞0.999 |
| Negative | | 0 | 2 | |  |
| **Adjuvant therapy** | |  |  | |  |
| Yes | | 2 | 14 | | 0.314 |
| No | | 1 | 1 | |  |
| **Student's t-test**: Age, Tumor volume, Extent of resection. **chi-square/Fisher’s exact test:** Gender, MGMT promoter methylation, EGFR Amplification, TERT promoter mutation, Adjuvant therapy. The pathological results of some cases are missing. | | | | | |

| **Supplementary Table 9. Demographic and Clinical Characteristics of Language type IV (Retrospective Study)** | | | | | |
| --- | --- | --- | --- | --- | --- |
| **Characteristics** | **Awake craniotomy group** | | | **Standard craniotomy group** | ***P* value** |
| **Gender** | |  |  | |  |
| Male | | 2 | 7 | | ＞0.999 |
| Female | | 2 | 5 | |  |
| **Age (years)** | | 36.3 ± 4.4 | 45.1 ± 3.2 | | 0.154 |
| **Tumor volume (ml)** | | 49.0 ± 15.9 | 56.8 ± 8.1 | | 0.683 |
| **Extent of resection (%)** | | 79.1 ± 8.1 | 71.9 ± 6.6 | | 0.511 |
| **MGMT promoter methylation** | |  |  | |  |
| Positive | | 2 | 8 | | ＞0.999 |
| Negative | | 1 | 3 | |  |
| **EGFR Amplification** | |  |  | |  |
| Positive | | 2 | 2 | | 0.467 |
| Negative | | 0 | 2 | |  |
| **TERT promoter mutation** | |  |  | |  |
| Positive | | 1 | 5 | | ＞0.999 |
| Negative | | 2 | 6 | |  |
| **Adjuvant therapy** | |  |  | |  |
| Yes | | 4 | 11 | | ＞0.999 |
| No | | 0 | 1 | |  |
| **Student's t-test**: Age, Tumor volume, Extent of resection. **chi-square/Fisher’s exact test:** Gender, MGMT promoter methylation, EGFR Amplification, TERT promoter mutation, Adjuvant therapy. The pathological results of some cases are missing. | | | | | |

| **Supplementary Table 10. Demographic and Clinical Characteristics of Language type V (Retrospective Study)** | | | | | |
| --- | --- | --- | --- | --- | --- |
| **Characteristics** | **Awake craniotomy group** | | | **Standard craniotomy group** | ***P* value** |
| **Gender** | |  |  | |  |
| Male | | 10 | 7 | | 0.761 |
| Female | | 5 | 6 | |  |
| **Age (years)** | | 44.0 ± 3.2 | 49.1 ± 3.8 | | 0.320 |
| **Tumor volume (ml)** | | 45.6 ± 9.9 | 45.6 ± 7.3 | | ＞0.999 |
| **Extent of resection (%)** | | 78.4 ± 5.3 | 78.3 ± 4.3 | | 0.989 |
| **MGMT promoter methylation** | |  |  | |  |
| Positive | | 6 | 5 | | ＞0.999 |
| Negative | | 7 | 6 | |  |
| **EGFR Amplification** | |  |  | |  |
| Positive | | 4 | 4 | | 0.319 |
| Negative | | 9 | 2 | |  |
| **TERT promoter mutation** | |  |  | |  |
| Positive | | 7 | 8 | | 0.423 |
| Negative | | 6 | 3 | |  |
| **Adjuvant therapy** | |  |  | |  |
| Yes | | 14 | 12 | | ＞0.999 |
| No | | 1 | 1 | |  |
| **Student's t-test**: Age, Tumor volume, Extent of resection. **chi-square/Fisher’s exact test:** Gender, MGMT promoter methylation, EGFR Amplification, TERT promoter mutation, Adjuvant therapy. The pathological results of some cases are missing. | | | | | |

| **Supplementary Table 11. Demographic and Clinical Characteristics of the Cohort Study Population** | | | | | |
| --- | --- | --- | --- | --- | --- |
| **Characteristics** | **Awake craniotomy group** | | | **Standard craniotomy group** | ***P* value** |
| **Gender** | |  |  | |  |
| Male | | 6 | 6 | | 0.743 |
| Female | | 7 | 9 | |  |
| **Age (years)** | | 58.9 ± 3.1 | 50.9 ± 3.9 | | 0.302 |
| **Tumor volume (ml)** | | 36.5 ± 6.0 | 54.1 ± 7.9 | | 0.240 |
| **Extent of resection (%)** | | 89.7 ± 4.3 | 85.4 ± 4.0 | | 0.822 |
| **MGMT promoter methylation** | |  |  | |  |
| Positive | | 7 | 8 | | 0.978 |
| Negative | | 6 | 7 | |  |
| **TERT promoter mutation** | |  |  | |  |
| Positive | | 7 | 8 | | ＞0.999 |
| Negative | | 5 | 7 | |  |
| **EGFR Amplification** | |  |  | |  |
| Positive | | 5 | 6 | | 0.934 |
| Negative | | 8 | 9 | |  |
| **Chemical therapy** | |  |  | |  |
| Yes | | 12 | 14 | | 0.916 |
| No | | 1 | 1 | |  |
| **Radiation therapy** | |  |  | |  |
| Yes | | 11 | 14 | | 0.457 |
| No | | 2 | 1 | |  |
| **Intraoperative seizure** | |  |  | |  |
| Yes | | 3 | - | | - |
| No | | 10 | - | |  |
| **Student's t-test**: Age, Tumor volume, Extent of resection. **chi-square/Fisher’s exact test:** Gender, MGMT promoter methylation, IDH status, TERT promoter mutation, EGFR Amplification, Chemical therapy, Radiation therapy. | | | | | |

**Supplementary Table 12. Change in the Fugl–Meyer Assessment score in a prospective cohort**

| **Number** | **Group** | **3 Days** | **14 Days** | **3 Months** |
| --- | --- | --- | --- | --- |
| 1 | Awake craniotomy | 10 | 60 | 90 |
| 2 | Awake craniotomy | 13 | 40 | 92 |
| 3 | Awake craniotomy | 36 | 85 | 90 |
| 4 | Awake craniotomy | 80 | 95 | 100 |
| 5 | Awake craniotomy | 85 | 95 | 100 |
| 6 | Awake craniotomy | 80 | 94 | 95 |
| 7 | Awake craniotomy | 85 | 98 | 100 |
| 8 | Awake craniotomy | 78 | 92 | 100 |
| 9 | Awake craniotomy | 82 | 94 | 100 |
| 10 | Awake craniotomy | 75 | 92 | 100 |
| 11 | Awake craniotomy | 74 | 90 | 95 |
| 12 | Awake craniotomy | 84 | 90 | 95 |
| 13 | Awake craniotomy | 80 | 85 | 90 |
| 14 | Standard craniotomy | 0 | 10 | 36 |
| 15 | Standard craniotomy | 15 | 40 | 85 |
| 16 | Standard craniotomy | 80 | 85 | 90 |
| 17 | Standard craniotomy | 12 | 10 | 55 |
| 18 | Standard craniotomy | 0 | 10 | 45 |
| 19 | Standard craniotomy | 65 | 80 | 90 |
| 20 | Standard craniotomy | 80 | 90 | 100 |
| 21 | Standard craniotomy | 0 | 15 | 60 |
| 22 | Standard craniotomy | 85 | 90 | 100 |
| 23 | Standard craniotomy | 82 | 90 | 100 |
| 24 | Standard craniotomy | 85 | 90 | 100 |
| 25 | Standard craniotomy | 78 | 85 | 95 |
| 26 | Standard craniotomy | 18 | 78 | 90 |
| 27 | Standard craniotomy | 10 | 60 | 90 |
| 28 | Standard craniotomy | 13 | 40 | 92 |

**Supplementary Table 13. Change in the muscle strength in a prospective cohort**

| **Number** | **Group** | **3 Days** | **14 Days** | **3 Months** |
| --- | --- | --- | --- | --- |
| 1 | Awake craniotomy | 1 | 3 | 5 |
| 2 | Awake craniotomy | 1 | 2 | 5 |
| 3 | Awake craniotomy | 2 | 5 | 5 |
| 4 | Awake craniotomy | 5 | 5 | 5 |
| 5 | Awake craniotomy | 5 | 5 | 5 |
| 6 | Awake craniotomy | 5 | 5 | 5 |
| 7 | Awake craniotomy | 5 | 5 | 5 |
| 8 | Awake craniotomy | 5 | 5 | 5 |
| 9 | Awake craniotomy | 5 | 5 | 5 |
| 10 | Awake craniotomy | 5 | 5 | 5 |
| 11 | Awake craniotomy | 5 | 5 | 5 |
| 12 | Awake craniotomy | 5 | 5 | 5 |
| 13 | Awake craniotomy | 5 | 5 | 5 |
| 14 | Standard craniotomy | 1 | 5 | 5 |
| 15 | Standard craniotomy | 2 | 5 | 5 |
| 16 | Standard craniotomy | 0 | 1 | 2 |
| 17 | Standard craniotomy | 1 | 2 | 5 |
| 18 | Standard craniotomy | 5 | 5 | 5 |
| 19 | Standard craniotomy | 1 | 1 | 2 |
| 20 | Standard craniotomy | 0 | 1 | 3 |
| 21 | Standard craniotomy | 3 | 5 | 5 |
| 22 | Standard craniotomy | 5 | 5 | 5 |
| 23 | Standard craniotomy | 0 | 1 | 2 |
| 24 | Standard craniotomy | 5 | 5 | 5 |
| 25 | Standard craniotomy | 5 | 5 | 5 |
| 26 | Standard craniotomy | 5 | 5 | 5 |
| 27 | Standard craniotomy | 5 | 5 | 5 |
| 28 | Standard craniotomy | 1 | 5 | 5 |

**Supplementary Table 14. Multivariable Cox regression analysis of overall survival in prospective cohort.**

| Demographic and clinical characteristics | Univariate analysis | | | |
| --- | --- | --- | --- | --- |
|  | *p* value | | Hazard Ratio | 95% CI |
| Age | | 0.142 | - | - |
| Tumor volume | | 0.779 | - | - |
| Extent of resection | | 0.219 | - | - |
| Surgical approach (reference: Awake craniotomy) | | 0.327 | - | - |
| EGFR Amplification (reference: negative) | | 0.544 | - | - |
| MGMT promoter methylation (reference: negative) | | 0.184 | - | - |
| TERT promoter mutation | | 0.792 |  |  |
| PFS = progression-free survival; Multivariate analysis method: forward, LR. | | | | |

**Supplementary figure：**

**Supplementary figure 1**：Flow diagram of the progress through the phases of a prospective cohort of GBM involving the precentral gyrus.

Allocation Phase: In the awake craniotomy group, two patients did not receive the allocated intervention. The reason was that these patients experienced discomfort at the initiation of the awake anesthesia process, leading to conversion to the standard craniotomy procedure.

Follow-up Phase: In the standard craniotomy group, one patient was lost to follow-up for the primary outcome. The reason was that the contact telephone numbers for the patient and their family were unreachable, despite repeated attempts.

**Supplementary figure 2** Motor-related tumor subtypes diagram. **A:** Axial plane; **B:** Sagittal plane; **C:** Coronal plane.

**Blue tract:** corticospinal tract; **Tumor location:** (I) Tumors involving the precentral gyrus; (II) Tumors involving premotor or supplementary motor regions without precentral gyrus infiltration; (III) Tumors adjacent to or invading the posterior limb of the internal capsule.

**Supplementary figure 3** Language-related tumor subtypes diagram. **A:** Sagittal plane with brain mask; **B:** Sagittal plane showing insular lobe and type IV; **C:** Sagittal plane showing Superior Longitudinal Fasciculus / Arcuate Fasciculus (SLF/AF) invasion in type I, II, and III.

**Orange tract:** SLF/AF; **Tumor location:** (I) Tumors involving inferior frontal lobe or precentral gyrus; (II) Tumors involving posterior central gyrus or supramarginal gyrus lesions superior to the lateral fissure; (III) Tumors involving the posterior region of the superior or middle temporal gyri or the supramarginal gyrus (below the lateral fissure level); (IV) predominantly insular lobe infiltration.
